# Brain functions and cognition on transient insulin deprivation in type 1 diabetes

**DOI:** 10.1172/jci.insight.144014

**Published:** 2021-03-08

**Authors:** Ana L. Creo, Tiffany M. Cortes, Hang Joon Jo, Andrea R.S. Huebner, Surendra Dasari, Jan-Mendelt Tillema, Aida N. Lteif, Katherine A. Klaus, Gregory N. Ruegsegger, Yogish C. Kudva, Ronald C. Petersen, John D. Port, K. Sreekumaran Nair

**Affiliations:** 1 Division of Pediatric Endocrinology and Metabolism,; 2 Division of Endocrinology, Diabetes, Metabolism, and Nutrition,; 3 Department of Radiology,; 4 Division of Neurocognitive Disorders,; 5 Division of Biomedical Statistics and Informatics,; 6 Division of Child and Adolescent Neurology,; 7 Division of Behavioral Neurology, and; 8 Division of Neuroradiology, Mayo Clinic, Rochester, Minnesota, USA.

**Keywords:** Endocrinology, Diabetes, Insulin, Memory

## Abstract

**BACKGROUND:**

Type 1 diabetes (T1D) is a risk factor for dementia and structural brain changes. It remains to be determined whether transient insulin deprivation that frequently occurs in insulin-treated individuals with T1D alters brain function.

**METHODS:**

We therefore performed functional and structural magnetic resonance imaging, magnetic resonance spectroscopy, and neuropsychological testing at baseline and following 5.4 ± 0.6 hours of insulin deprivation in 14 individuals with T1D and compared results with those from 14 age-, sex-, and BMI-matched nondiabetic (ND) participants with no interventions.

**RESULTS:**

Insulin deprivation in T1D increased blood glucose, and β-hydroxybutyrate, while reducing bicarbonate levels. Participants with T1D showed lower baseline brain N-acetyl aspartate and myo-inositol levels but higher cortical fractional anisotropy, suggesting unhealthy neurons and brain microstructure. Although cognitive functions did not differ between participants with T1D and ND participants at baseline, significant changes in fine motor speed as well as attention and short-term memory occurred following insulin deprivation in participants with T1D. Insulin deprivation also reduced brain adenosine triphosphate levels and altered the phosphocreatine/adenosine triphosphate ratio. Baseline differences in functional connectivity in brain regions between participants with T1D and ND participants were noted, and on insulin deprivation further alterations in functional connectivity between regions, especially cortical and hippocampus-caudate regions, were observed. These alterations in functional connectivity correlated to brain metabolites and to changes in cognition.

**CONCLUSION:**

Transient insulin deprivation therefore caused alterations in executive aspects of cognitive function concurrent with functional connectivity between memory regions and the sensory cortex. These findings have important clinical implications, as many patients with T1D inadvertently have periods of transient insulin deprivation.

**TRIAL REGISTRATION:**

ClinicalTrials.gov NCT03392441.

**FUNDING:**

Clinical and Translational Science Award (UL1 TR002377) from the National Center for Advancing Translational Science; NIH grants (R21 AG60139 and R01 AG62859); the Mayo Foundation.

## Introduction

There is a growing interest in the effects of type 1 diabetes (T1D) on brain development, cognition, and dementia, especially as the incidence and life expectancy of patients with diabetes increases (1–4). Although the Diabetes Complications and Control Trial established a substantial reduction in microvascular complications, uncertainty remains on the impact of glycemic control and dementia (5). Recent work highlights the effects of chronic glucose excursions and variability on neuroanatomy and cognitive function (2, 4, 6, 7). However, while past studies explored chronic dysglycemia (8–10) and the acute and selective effect of hyperglycemia in the presence of insulin (11), little is known about the effect of acute periods of insulin deficiency and resulting altered metabolic milieu on the brain (12). Although variability in insulin levels has no effect on brain glucose uptake in humans (13), preclinical studies in rodents indicate that insulin deprivation alters brain oxidative capacity and adenosine triphosphate (ATP) generation (14). As neuronal conduction is ATP dependent (15), it remains unknown whether insulin deprivation has a detrimental effect on brain functional connectivity (FC) and cognitive performance in people with T1D.

Deliberate omission of insulin for weight loss has been reported in young individuals with T1D (16, 17). With the rapidly growing use of continuous subcutaneous inulin infusion pumps, it is common to have periods of insulin deprivation associated with missed insulin boluses, device malfunctions, or suspensions, and likewise, people on multiple daily insulin injections also miss occasional injections, resulting in transient insulin deprivation (18–20). Although the adverse brain effects of acute hypoglycemia while on insulin treatment in T1D has been well described (21, 22), the effects of acute insulin deprivation on the brain and cognition remain to be determined.

Here we sought to determine the effect of acute insulin deprivation on brain metabolites, including ATP levels, FC, and cognition. We hypothesized that the disruptions in oxidative phosphorylation/brain energy pathways resulting from insulin deprivation would alter brain FC and cognition. We assessed whether these biochemical and functional measurements were altered in participants with T1D in comparison with nondiabetic (ND) participants using brain ^1^H and ^31^P magnetic resonance spectroscopy (MRS) and resting-state functional magnetic resonance imaging (rs-fMRI) as well as cognitive testing.

## Results

### Insulin deprivation, as anticipated, caused substantial biochemical changes in participants with T1D.

**Figure 1:**
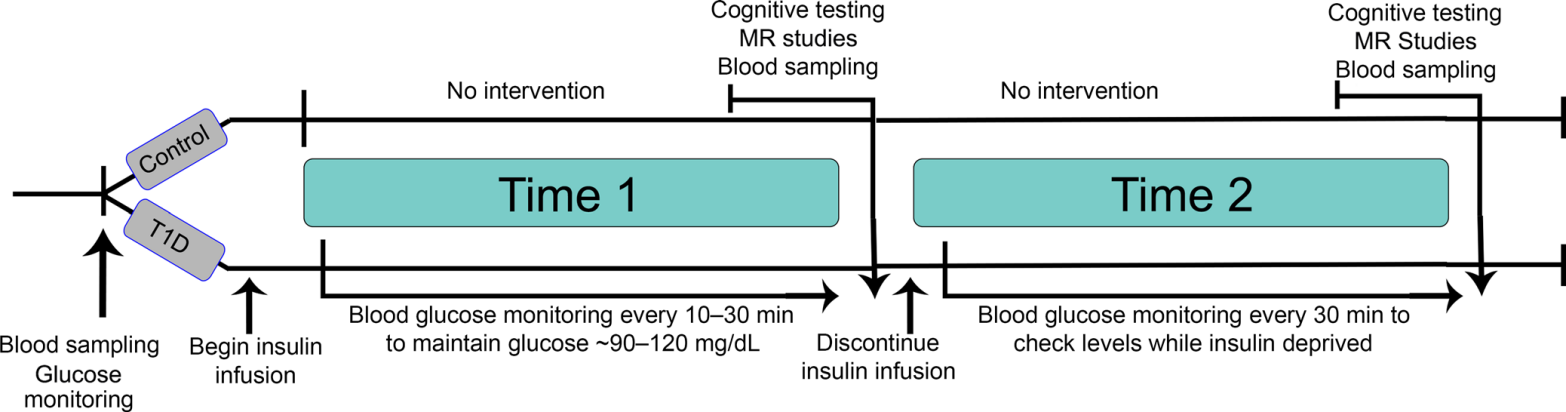
Study design. Both study groups (nondiabetic [ND] and T1D) started the study with a fasting blood sampling. Participants with T1D were then started on a continuous insulin infusion titrated to maintain blood glucose between 5 and 6.7 mmol/l (Time 1). Following a 3-hour insulin infusion in the T1D group or the corresponding time course for the ND group, participants had cognitive testing. Following this, the participants were taken to radiology, and MR studies were performed. The insulin infusion was continued during both cognitive testing and MR studies. Then, blood sampling was performed, and immediately after the insulin infusion was discontinued in the participants with T1D. Four hours following after a period of insulin withdrawal (Time 2), or at the corresponding time course for the control participants, the cognitive testing (4–4.5 hours) and then MR studies were performed (5 ± 0.6 hours). Time 1 represents the insulin-treated period in T1D, and Time 2 represents the period following insulin deprivation in T1 D. Both times represent no intervention but specifically address time-related changes in the ND group.

The study was designed to address whether transient insulin deprivation alters brain function (Figure 1). We compared the participants with T1D (age range, 14–28 years) to ND (age range, 16–29 years) participants who were matched for age, sex, and BMI. We did not include older participants with T1D to delineate the effects of diabetes from age-related brain changes. Mean glycated hemoglobin (HbA_1c_) (6.7%–8.8% in T1D vs. 4.3%–5.5% in ND groups, respectively) and Time 1 plasma glucose (7.68 ±.67 mmol/L in T1D vs. 5.15 ± 0.12 in ND) were different (*P* < 0.001) (Table 1). All participants were studied following an overnight fast. In participants with T1D, we administered insulin intravenously for 3 hours to maintain stable plasma glucose between 5 and 6.67 mmol/L by titrating the insulin infusion rates every 15–30 minutes, while ND participants received no insulin during the same period (Time 1). In response to discontinuing the insulin infusion for 5.4 ± 0.6 hours, plasma glucose in participants with T1D increased to an average of 14.4 mmol/L, which was higher than that in ND participants, 4.72 mmol/L, at the same time (*P* < 0.001) (Table 1). As expected, *β*-hydroxybutyrate (BOHB) concentrations were not different at Time 1 between participants with T1D and ND participants, but they significantly increased at Time 2 (following insulin deprivation) in participants with T1D but not in ND participants, rendering BOHB to be higher in the T1D group than the ND group (*P* < 0.001) (Table 1). Similarly, serum osmolality was also higher at Time 2 compared with Time 1 in the T1D group and was higher than ND participants who had no change during Time 1 and Time 2. At Time 2, bicarbonate fell in the T1D group, while there was no significant change in the ND group (Table 1). All of these changes are consistent with insulin deficiency in T1D.

### Effect of insulin deprivation on cognitive function.

**Figure 2:**
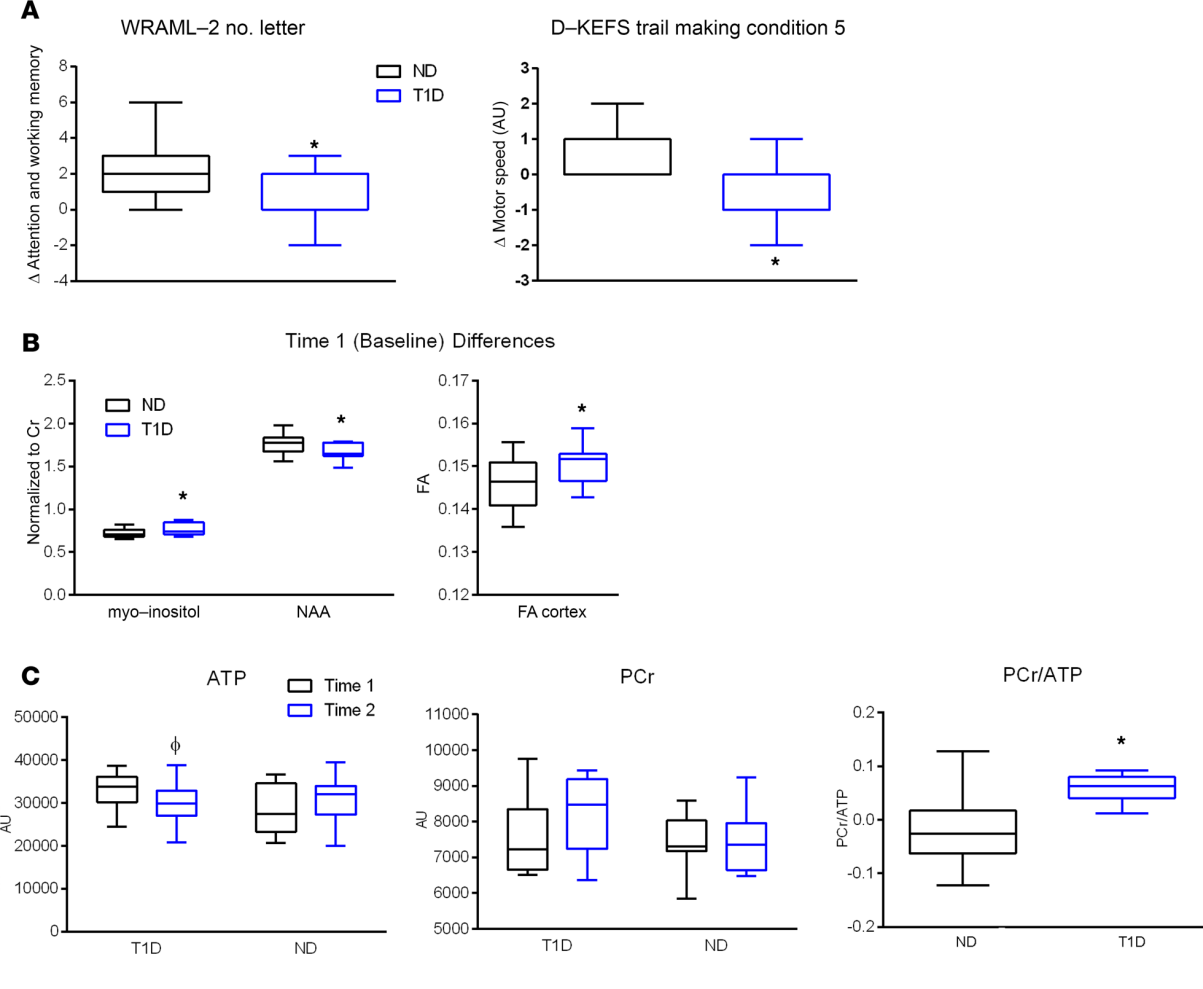
Cognitive test results showing changes in participants with type 1 diabetes from treatment with insulin (Time 1) to the insulin-deprived state (time 2) in comparison with the same period in participants without diabetes. (**A**) Compared with participants without diabetes (ND), participants with type 1 diabetes (T1D) showed significantly poorer attention and working memory based on the Wide Range Assessment of Memory and Learning-2 (WRAML-2) Number Letter subtest (*P < 0.001) and fine motor speed based on Delis-Kaplan Executive Function System (D-KEFS) Trail Making (*P < 0.02). (**B**) Baseline differences in myo-inositol (*P < 0.05), N-acetyl aspartate (NAA) (*P < 0.05), and cortical fractional anisotropy (FA) (*P < 0.05) between the T1D group and ND controls; none of them were significantly altered by insulin deprivation (data not shown). (**C**) Total adenosine triphosphate (ATP) levels that significantly decreased on insulin deprivation (Time 1 to Time 2) in participants with T1D (*P* < 0.04) and phosphocreatine (PCr) showed no significant changes. The ratio of PCr to ATP increased in the T1D group (*P* < 0.03).

We sought to determine whether transient insulin deprivation has any adverse effect on cognitive function. There were no differences between the participants with T1D and ND participants on any study measures, including those of memory, verbal and nonverbal executive function, attention, or short-term memory at Time 1 (obtained while participants with T1D were on insulin infusion). However, at Time 2 (following discontinuation of insulin infusion in participants with T1D), the T1D group performed poorly compared with participants ND in 2 areas of cognitive function testing. The T1D group showed slower fine motor speed on Delis-Kaplan Executive Function System (D-KEFS) Condition 5 at Time 2 compared with the ND group (Figure 2A). Also at Time 2, the T1D group showed less benefit from repeat exposure to tests of attention and working memory compared with ND participants. With the exception of the Ray Auditory Verbal Learning Test (RAVLT), there were no performance differences between adults and adolescents, and so the results were pooled for analysis. On the RAVLT memory test, adolescents in both the T1D and ND groups did especially poorly at Time 2 compared with Time 1. Adults with T1D and ND adults showed a decline in performance at Time 2 (*P* < 0.02), which was driven by the especially poor performance of ND adults. The overall decline in RAVLT may indicate the effect of fasting in adolescents and ND adults (Supplemental Table 1; supplemental material available online with this article; https://doi.org/10.1172/jci.insight.144014DS1).

### Changes in brain metabolites by proton MRS data.

Brain lactate concentration was low, at below detectable levels, in both participants with T1D and ND participants. However, at Time 1, the T1D group had lower N-acetyl acetate/creatine (NAA/Cr), while having higher myo-inositol/Cr (mI/Cr) than in the ND group. Neither NAA/Cr nor mI/Cr changed from Time 1 to Time 2 (Figure 2B).

### Phosphorus MRS data showing reduced ATP levels and increase of phosphocreatine/ATP.

We hypothesized that ATP levels would decrease from Time 1 to Time 2 in participants with T1D due to a shift to a low oxidative capacity in the setting of insulin deficiency, as noted in an animal model (14). We did indeed find that ATP levels decreased from Time 1 to Time 2 in participants with T1D compared with ND participants, while phosphocreatine (PCr) did not change in either group (Figure 2C). An increase in PCr/ATP occurred in the T1D group from Time 1 to Time 2 compared with the ND group (Figure 2C).

### Alterations in rs-fMRI FC following insulin deprivation.

**Figure 3:**
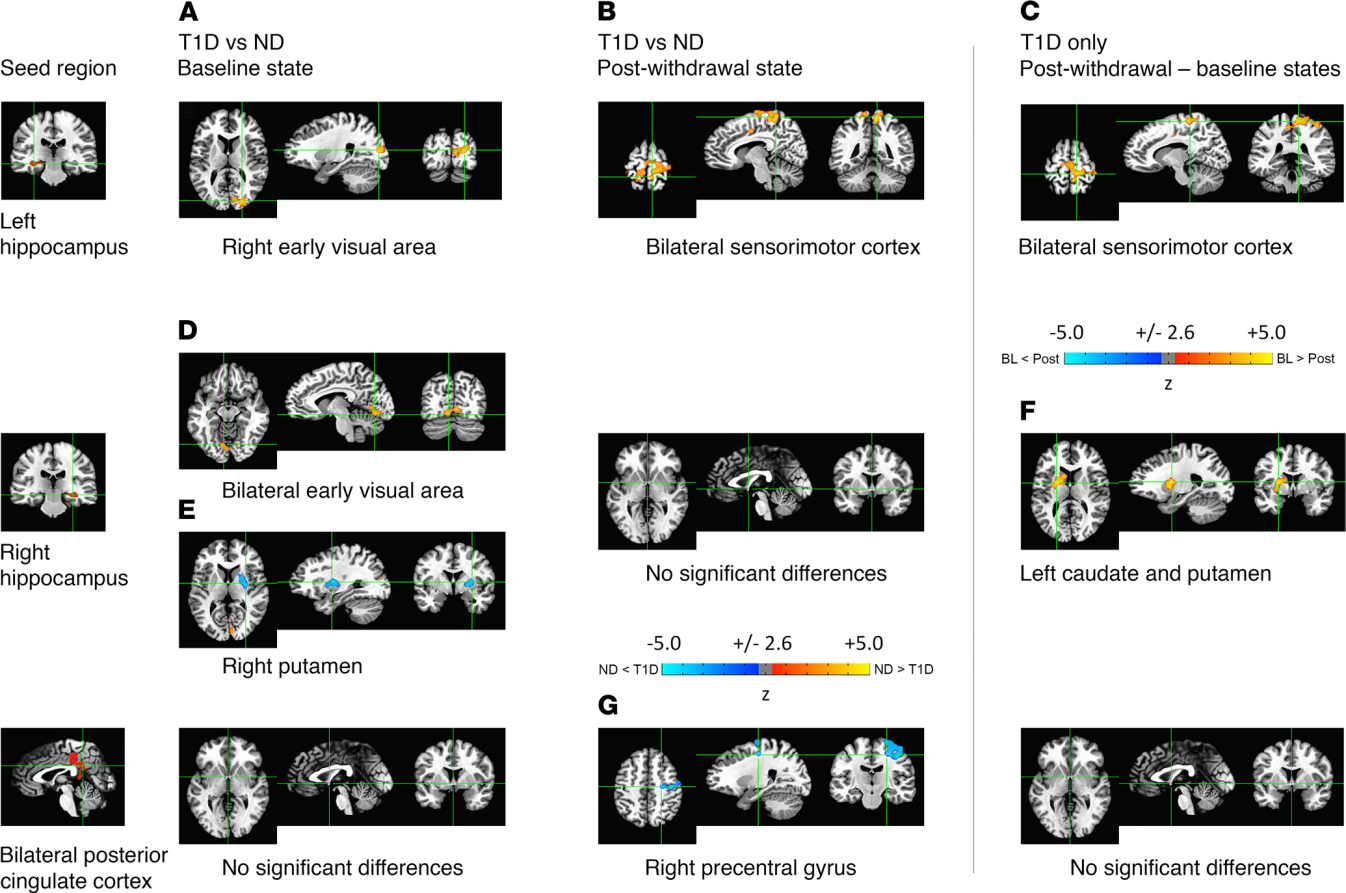
Seed-based functional connectivity maps. Seed masks were extracted from the FreeSurfer parcellation of the N27 brain template. Three seed regions reached statistical significance (*P* < 0.01) (left and right hippocampi and bilateral posterior cingulate cortex). The functional connectivity (FC) of these seed regions was higher (yellow) or lower (blue) compared with 6 different brain regions (Table 2). At baseline (left column), there was appreciably higher FC in nondiabetic (ND) participants relative to that in participants with type 1 diabetes (T1D) (**A**) between the left hippocampus and the right early visual area and (**D**) between the right hippocampus and bilateral early visual areas. (**E**) In contrast, there was significantly (*P* < 0.01) lower FC between the right hippocampus and right putamen in ND participants. There were no significant FC differences with the bilateral posterior cingulate cortex in the baseline state. Following insulin deprivation (middle column), there was substantially higher FC in ND participants relative to that in participants with T1D (**B**) between the left hippocampus and bilateral sensorimotor cortices but (**G**) lower FC between both posterior cingulate cortices and the right precentral gyrus. There were no significant FC differences with the right hippocampus in the postwithdrawal state. Finally, assessing changes between the postwithdrawal and baseline states in participants with T1D (right column), (**C**) there was considerably decreasing FC between the left hippocampus and bilateral sensorimotor cortices and (**F**) the right hippocampus and the left caudate/putamen following insulin withdrawal, indicating that insulin deprivation adversely affected FC between these regions in participants with T1D. No significant FC differences with the bilateral posterior cingulate cortex were found in participants with T1D between the baseline and postwithdrawal states. Note that the FC clusters for the bilateral sensorimotor cortex identified in **B** and **C** are similar but not identical (see Table 2).

rs-fMRI analysis was performed, setting significance at α < 0.01,|t| > 2.576, *P* < 0.01 and a minimum cluster size of more than 119 voxels (23, 24). We found a total of 6 functionally connected brain regions originating from 3 seed regions (Figure 3, numerical details are provided in Table 2).

At Time 1, higher FC was demonstrated between the left hippocampus and the right early visual area (Figure 3A) in the ND group relative to the T1D group (Figure 3B), indicating that even with insulin treatment there were abnormalities that existed in the T1D group. In the T1D group, when comparing Time 2 versus Time 1, the FC between the left hippocampus and bilateral sensorimotor cortex was also lower (Figure 3C) than that in the ND group, showing that insulin deprivation was associated with lower FC. At Time 1, FC between the right hippocampus and right and left early visual areas was higher in the ND group but lower in the right putamen (Figure 3, D and E). The ND group also had higher FC between the right hippocampus to left caudate and putamen (Figure 3F). The differences in FC between Time 1 and Time 2 of posterior cingulate cortex and right precentral gyrus were lower in the ND group than in the T1D group. (Figure 3G).

### Correlations between cognitive function and MRS/rs-fMRI findings.

Correlations between the seed areas and cognitive testing results, as well as MRS metabolite findings, were assessed (Table 3). Choline (Cho), an important neurotransmitter essential for brain functions, including memory, correlated to FC between the hippocampus and sensorimotor cortex. mI and NAA, which are abundant in the brain, correlated to FC between the hippocampus and early visual area at Time 1; PCr was also correlated to FC between posterior cingulate cortex and precentral gurus. In the diabetic group at Time 1, glutathione/Cr (GSH/Cr) was correlated to hippocampus and caudate and putamen regions, and a negative correlation with GSH/Cr was noted (Time 1 to Time 2) between the hippocampus and sensory motor cortex unlike in ND. In the T1D group at Time 2, GSH/Cr was higher, likely representing an oxidative stress response. There were significant correlations between the hippocampal region and sensorimotor cortex (Time 2 to Time 1) and mI/Cr levels.

FC between the hippocampus and visual areas, the hippocampus and sensorimotor cortex, and the hippocampus and caudate and putamen was significantly either positively or negatively correlated in both diabetic and ND groups. In the diabetic group, FC (Time 2 to Time 1) in the hippocampus and sensorimotor cortex, the hippocampus and early visual area, and the hippocampus and putamen was negatively correlated to important cognitive changes (Table 3).

## Discussion

The current study demonstrates significant differences in proton MRS-based biomarkers of neuronal health, such as NAA, mI, and cortical fractional anisotropy, in the T1D group while on insulin treatment in comparison with ND controls. Further, phosphorus MRS demonstrated alterations in bioenergetics, as shown by a decline in ATP levels and alteration the PCr/ATP ratio during insulin deprivation in the T1D group. Transient insulin deprivation (mean 5.4-hour duration) in the T1D group compared with the ND group also resulted in diminished executive function, including attention, short-term memory, and fine motor speed. Although the T1D group had baseline differences in FC, more important changes occurred following insulin deprivation, especially between the hippocampus-caudate-putamen regions and the sensory motor and early visual areas. We also noted significant correlations in regional brain FC to cognitive function, neurometabolites, and energy parameters.

The rs-fMRI findings in the current study are intriguing from a number of perspectives. First, FC cluster analysis identified seed areas not reported in any major functional network (e.g., default mode network) (25). This identification is likely due to methodologic differences between seed-based analysis techniques and nonconstrained, predominantly independent component-based analyses (26). Of interest, other seed-based studies also reported FC correlations of the hippocampus to other regions such as the medial temporal lobe (27, 28). Furthermore, Griffanti et al. (29) performed seed-based FC correlations of the basal ganglia and found a significant negative correlation between age and the basal ganglia FC to other brain structures. In the current study, FC between different regions involved in memory differed between ND and diabetic groups. For example, lower FC between the left hippocampus and the right early visual area (Figure 3A) and between the right hippocampus and both early visual areas (Figure 3D) were noted in the T1D group relative to the ND group (Table 2). In contrast, there was higher FC between the right hippocampus and right putamen (Figure 3E). After insulin deprivation, the most altered FCs were in the hippocampus-caudate to sensorimotor cortexes (Figure 3 and Table 2), which are involved in memory. Overall, our data also suggest that in the T1D group at baseline and during insulin deprivation the accumulation of deleterious neurometabolites, including energy metabolites, altered FC, which then contributes to cognitive changes. Further studies with larger samples will be helpful to validate these important findings.

We noted that following insulin deprivation, the T1D group failed to demonstrate expected learning upon repeat cognitive testing in executive function domains. The T1D group had slowed fine motor speed following insulin deprivation, whereas the ND group increased fine motor speed during the same period. The T1D group also benefited less from repeated exposure to tests of attention and working memory compared with the ND group. The alertness and attention required for success on this task are mediated by an interaction of brain stem–diencephalic structures, which communicate through several pathways to structures involved in memory and sensorimotor regulations (30). Of interest, all of the above areas causing cognitive decline occurred in the participants with T1D during insulin deprivation in brain regions with abundant insulin receptors (31).

While chronic poor glycemic control has been associated with changes in the hippocampus and memory, it is unknown whether transient insulin deficiency worsens brain functioning (32, 33). The present study did not isolate insulin deficiency from hyperglycemia and other metabolic changes, including an increase in BOHB, nonesterified fatty acids, and amino acids, as well as glucagon levels (34, 35). However, it remains plausible that insulin deficiency independently produces neurometabolic changes. There is differential distribution throughout the brain of insulin and IGF1 receptors and their posttranslational signaling partners (31). Moreover, intranasal insulin administration in diabetic mice increased brain insulin concentrations without any systemic glucose concentration but activated downstream signaling, such as PI3K/Akt and GDK3β, and caused a notable effect on mitochondrial biogenesis and function in brain regions such as the hippocampus (14). The above results support a hypothesis that insulin directly acts on brain regions involved in memory by increasing energy metabolism. Some of the proposed downstream pathways of insulin receptors, such as the PI3K/Akt, mTORC1, GDK3β, and FoxO group of transcription factors, play crucial roles in maintaining normal brain function (14, 36–39). Likewise, insulin receptors directly interact with the MAP kinase cascades known to affect memory (40–42). Insulin is a key regulator of mitochondrial biogenesis and function in multiple tissues (14, 43), including skeletal muscle, liver, and brain. In diabetic mice (44) and humans with T1D (35), insulin deprivation directly and substantially inhibited mitochondrial ATP production and detrimentally increased oxidative stress in skeletal muscle. However, the effect of insulin deprivation on brain energetics in mice was more subtle and surprisingly enhanced the endogenous antioxidant defense system, thus protecting the brain against oxidative stress, which appeared to be due to a beneficial effect of ketones and lactate (14). During transient insulin deprivation in the current study, a substantial BOHB elevation may have offered antioxidant defense and may explain why only subtle changes in brain energetics occurred. No comparable human studies, to our best knowledge, have explored brain changes related to transient insulin deprivation. Our study not only illuminates potential mechanisms driving longitudinal changes seen in FC and cognitive functioning–associated insulin deficiency and metabolic alterations, but also demonstrates the effects of acute insulin omission with important clinical implications on FC between brain regions and executive functions. The current study quantifies the neurologic changes associated with lapses in insulin administration commonly encountered in the growing number of people using insulin pumps (45). Results from the current study may inform further clinical practice and counseling for adults and children with T1D who are reliant upon continuous insulin infusion devices as well as self-administration of multiple daily insulin regimens.

In the current study of individuals with T1D, diabetes onset was variable, and no conclusion on the effect of diabetes duration on our results could be drawn. Previous studies have addressed structural and functional brain changes that occur on chronic poor glycemic control (22, 46). The current study demonstrated differences in MRS-based brain biomarkers of neuronal health, such as NAA, mI, and cortical fractional anisotropy, in the T1D group at the baseline state. As the above parameters did not change following insulin deprivation in the current study, the differences in the above brain metabolites that we observed between participants with T1D and ND participants represent potential suboptimal glycemic control of longer term. Although hyperinsulinemia with hypoglycemia also have adverse effects on the brain (21, 22), it has been shown that transient hypoglycemia does not alter energy-rich phosphometabolites in the brain (21). Moreover, it appears that during hypoglycemia brain extracts higher fraction of glucose than during normoglycemia, so that its metabolic needs can be maintained during modest hypoglycemia (13). In contrast, in the current study, we demonstrate that hypoinsulinemia with the concurrent metabolic changes altered brain energy metabolites. The current study measured static PCr and ATP levels representing the concentration of these metabolites within the cell but did not address the kinetics of these energy metabolites (47), which will require longer phosphorus MRS scans, with different saturation transfer pulses, that would not have been practical due to all the measurements we performed. Our focus was not only energy metabolites, but also acquiring other anatomical or functional imaging.

Our results must be interpreted within the context of several limitations. First, a larger sample size may have the ability to detect changes in brain volume associated with insulin deprivation. Second, as with the constraints of a human study, we were unable to further isolate the complex metabolic and hormonal changes occurring with insulin deprivation, including hyperglycemia, increased nonesterified fatty acids, and BOHB, amino acid, and glucagon concentrations as well as volume changes, all of which could have potential independent effects on the brain. Further studies are required to differentiate the effect of each of these variables associated with insulin deprivation and isolate the effect of insulin deprivation alone on brain. However, preclinical and early clinical studies clearly established the selective effect of insulin on brain metabolism, including cognition (14, 48, 49). We limited the insulin deprivation duration for participant safety and due to risk for diabetic ketoacidosis, particularly in the pediatric group, in which even after 6 hours, some adolescents had substantial metabolic derangements.

In conclusion, we found that transient insulin deprivation in individuals with T1D altered brain connectivity between areas of memory and sensorimotor as well as early visual regions concurrent with alterations in cognition in the domains of executive functions and fine motor speed and subtle changes in brain bioenergetics and metabolites. Further understanding the neurocognitive effects of transient insulin deprivation, as highlighted in this study, will have important clinical implications, given the growing number of people with diabetes and use of insulin pumps across the world.

## Methods

### Study participants.

Adults and adolescents with T1D were recruited from patients at Mayo Clinic, Rochester, Minnesota, and the surrounding Olmstead County, Minnesota, USA, community. Adult ND controls were healthy age-, sex-, and BMI- matched participants, while adolescent ND controls were same sex siblings of similar age (±1 year) or a friend of similar BMI and age (±6 months). All participants were screened with a detailed history, physical table examination, and biochemical profile. Exclusion criteria included diagnosed cognitive delay, attention-deficit/hyperactivity disorder, learning disabilities, dementia, psychiatric disease, cardiovascular and cerebrovascular diseases, peripheral neuropathy, renal disease, substance use, or obesity.

Participants with T1D needed to have a HbA_1c_ of less than 9%, BMI of 20–30 kg/m^2^ for adults and less than 95th percentile for adolescents, c-peptide of less than 1 ng/mL, and no evidence of active diabetic complications, including renal disease, peripheral vascular disease, and neuropathy.

The average diabetes duration for participants was 10.7 (± 1.7) years (range, 1–25 years).

### Study protocol.

Figure 1 outlines the study design. Before the study day, participants with T1D using insulin injections held long-acting insulin 36 hours to avoid any carryover effect, managing glucose with short-acting insulin corrections. Participants with T1D using insulin pumps continued on the pump until the study start day. All participants maintained typical sleep and exercise schedules the week prior to the study.

Participants were admitted after an overnight fast. Before Time 1 of the study, all participants had baseline blood biochemical measurements, including serum glucose, taken. After these baseline measurements, participants entered Time 1. Participants with T1D were transitioned to intravenous regular insulin infusion titrated to target blood glucose of 5–6.67 mmol/L. Titration was performed using point-of-care glucose devices, as transportable devices were necessary in the MRI suite and allowed frequent glucose monitoring every 15–30 minutes to ensure that each participant remained in range throughout the MRI study. Participants continued fasting with hydration from intravenous normal saline and water ad lib. Participants then underwent 30 minutes of cognitive testing followed by MRI scans as detailed below.

After Time 1, participants entered Time 2. During Time 2, participants with T1D underwent insulin deprivation and ND participants continued hydration. Following more than 4 hours in Time 2, cognitive testing and MRI studies were repeated while insulin remained off for participants with T1D, with a 6-hour maximal period of insulin deprivation, as there was some variance within the study day in how long it took each subject to complete all study tests. Final blood sampling was performed before insulin was restarted. Participants with T1D were observed until clinically stable and the blood glucose was less than 11.1mmol/L, at which time they ate a meal and were discharged. ND participants underwent similar laboratory, cognitive, and MRI studies at Time 1 and Time 2 without any insulin treatment or deprivation.

### Hormones and substrates.

Plasma glucoses were assayed in samples collected at baseline and following insulin deprivation enzymatically using the Roche Modular platform. Plasma c-peptide, bicarbonate, BOHB, and electrolyte profiles were measured as previously described (35).

### Cognitive testing.

All subjects underwent a cognitive battery before each MRI. This broad, multidimensional cognitive evaluation consisted of the RAVLT, the D-KEFS, the Trail Making Test, and the Number Letter subtest of the Wide Range Assessment of Memory and Learning (WRAML).

The RAVLT is a memory test assessing acquisition, learning rate, susceptibility to interference, and forgetting (50). The RAVLT has marginal/adequate test-retest reliability, with *r* values about 0.60–0.70 (51–53). The D-KEFS measures various aspects of executive functions (54). The Trail Making Test assesses visual scanning, mental sequencing, thinking flexibility, and motor speed. Test-retest reliability ranges from *r* = 0.38 to *r* = 0.77. The Verbal Fluency test of the D-KEFS assesses an individual’s ready access to verbal stores of information. Test-retest reliability ranges from *r* = 0.36 to *r* = 0.8. The Number Letter subtest of the WRAML measures attention and short-term memory (55). Median coefficient α reliability is *r* = 0.83. The combination of the above tests yielded a broad assessment of the subject’s cognitive function before and after insulin withdrawal.

### MRI data acquisition.

Brain imaging was performed on a single 3 Tesla Siemens Skyra equipped with a multinuclear option running VE11C software. Each participant was scanned twice on the same day using the same scan protocol. A 32-channel proton-only head coil was used for all MR imaging and proton MRS. A dual-tuned proton/phosphorus flex coil (Clinical MR Solutions; 16 × 18 cm diameter Helmholtz pair for proton, 12 cm diameter loop coil for phosphorus) was used for phosphorus MRS. A sagittal 3D Magnetization Prepared — Rapid Gradient Echo (MPRAGE) sequence with 0.7 mm isotropic voxels (TR, 2400 ms; TE, 2.57 ms; TI, 1100 ms, FA = 8 degrees) was acquired in order to obtain brain parcellations for subsequent rs-fMRI analyses.

To assess brain energy pathways, both proton and phosphorus MRS were performed in order to measure brain lactate (anaerobic metabolism) and ATP levels (aerobic metabolism). Proton MRS was acquired using the svs_se sequence with the voxel placed over the occipital lobes (TR, 2000 ms; TE, 30 ms; 128 averages). Phosphorus MRS was acquired using a multivoxel chemical shift imaging sequence (WIP 1071, qa_csi_fid_31P) applied such that the phosphorus loop was positioned over the occipital cortices. An axial 1.5 cm CSI slab was acquired to encompass the occipital cortices (TR, 1500 ms; TE, 30 ms; 16 × 16 matrix over a 240 cm FOV, 1.5 cm nominal isotropic voxels, 2 averages).

To assess FC, rs-fMRI imaging was acquired using an axial 2D echoplanar imaging sequence with 3 mm isotropic voxels (FA, 90 degrees; TE, 30 ms; TR, 3000 ms; no. slices, 52; 116 volumes; volumes; 5 minutes and 48 seconds). Participants were instructed to lie still in the scanner, but given no other instructions besides that.

### MRI data processing.

Single-voxel proton MRS data were processed using LCModel 6.3-1L (56). Spectra were visually inspected, and any corrupted spectra were excluded from the analysis. Besides lactate, Cho, NAA, glutamate, glutamine, glutamate-glutamine, GSH, and mI ratios expressed using Cr were also tabulated. All metabolite values with Cramér-Rao lower bound less than or equal to 20% were kept in the analysis. No attempt was made to perform cerebrospinal fluid volume correction; we report only ratios.

Multivoxel phosphorus magnetic resonance spectroscopic imaging data were reconstructed and quantified using jMRUI 6.0 (http://www.jmrui.eu). Spectra were preprocessed by (a) truncating the data to 768 points, then 0-filling to 1024 points; (b) apodizing with a 5 Hz Lorenzian; and (c) aligning the data such that the PCr peak was set to 0 Hz. Next, 2 voxels from occipital cortex were selected, extracted, and averaged together into a single free induction decay in order to reduce noise. Finally, as we were only interested in ATP and PCr levels, the average free induction decay was quantified using AMARES and a 4-metabolite basis set (PCr, γ-ATP, α-ATP, β-ATP) (52). The PCr/ATP ratio was calculated using the sum of the γ-ATP, α-ATP, and β-ATP peaks for the denominator.

MPRAGE data were analyzed using Freesurfer 6.0 (http://surfer.nmr.mgh.harvard.edu). MPRAGE images were converted to a Neuroimaging Informatics Technology Initiative volume using mri_convert, then processed using recon_all with manual inspection of data between each recon step. The Freesurfer N27 template (57) was then used to obtain cortical and subcortical parcellations for FC matrix calculations. Resting-state functional MRI data were motion-corrected and denoised by a well-established preprocessing pipeline in the NIH rs-fMRI analysis tool (AFNI package) as previously described (8, 9). Briefly, rs-fMRI data were despiked and corrected for physiologic noise, slice timing, head motion, and hardware artifacts. The corrected data were spatially smoothed with an isotropic Gaussian kernel (full-width-at-half-maximum at 6 mm) and then registered to the MPRAGE imaging (58) to get functional blood flow data for each cortical/subcortical brain region obtained from the N27 parcellation. Individual seed-based connectivity matrices for whole subjects were used for the group comparisons between diabetic and control groups and also for correlation analysis among FC strength, clinical outcomes, (behavioral scores) and metabolic data (proton and phosphorus metabolites) (10).

### Statistics.

We summarized measurements and paired differences using descriptive statistics. Data were inspected for normality, and transformations applied or nonparametric tests were used when necessary. A nonparametric robust analysis of variance-type statistic (F1-LD-F1 design in nparLD) was utilized to assess the effect of insulin deprivation in patients with T1D when compared with ND participants (59). Two-tailed *t* tests were used to compare the T1D group to the ND group. All statistical tests were 2-sided with a significance threshold of *P* ≤ 0.05. No corrections were made for multiple comparisons. As our subjects had a narrow age distribution, no corrections were made for age, as there was no need to eliminate age-related effects.

### Study approval.

Written informed consent or parental permission was obtained for all participants before the study. The Mayo Clinic Institutional Review Board approved these experiments.

## Author contributions

ALC and TMC conducted the study, wrote the manuscript, and researched the data. KSN designed and supervised the study and analysis of samples, researched and interpreted the data, and revised and edited manuscript. JDP supervised all MRI, MRS, and rs-fMRI and interpreted the data and contributed to the manuscript. HJJ performed the analysis of rs-fMRI data. ARH performed and analyzed neuropsychological tests, interpreted results, and contributed to the manuscript. SD performed statistical analysis. JMT and ANL contributed to the participant screening and selection in addition to reviewing manuscript. KAK and GNR offered valuable technical help and contributed to the manuscript. YCK offered clinical support and contributed to manuscript. RCP contributed to the manuscript.

## Supplementary Material

Supplemental data

Trial reporting checklists

ICMJE disclosure forms

## Figures and Tables

**Table 1:**
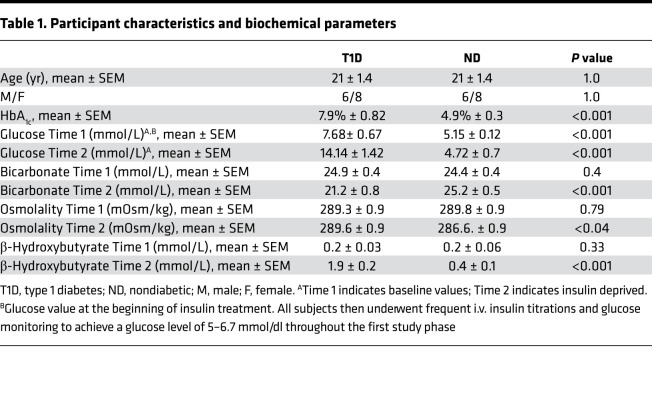
Participant characteristics and biochemical parameters

**Table 2:**
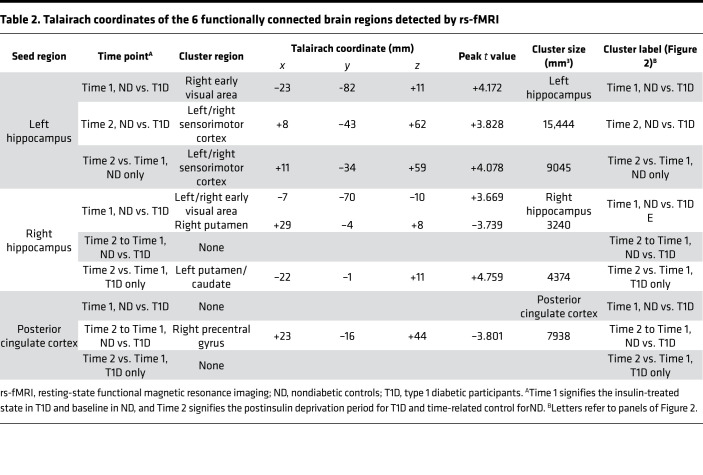
Talairach coordinates of the 6 functionally connected brain regions detected by rs-fMRI

**Table 3:**
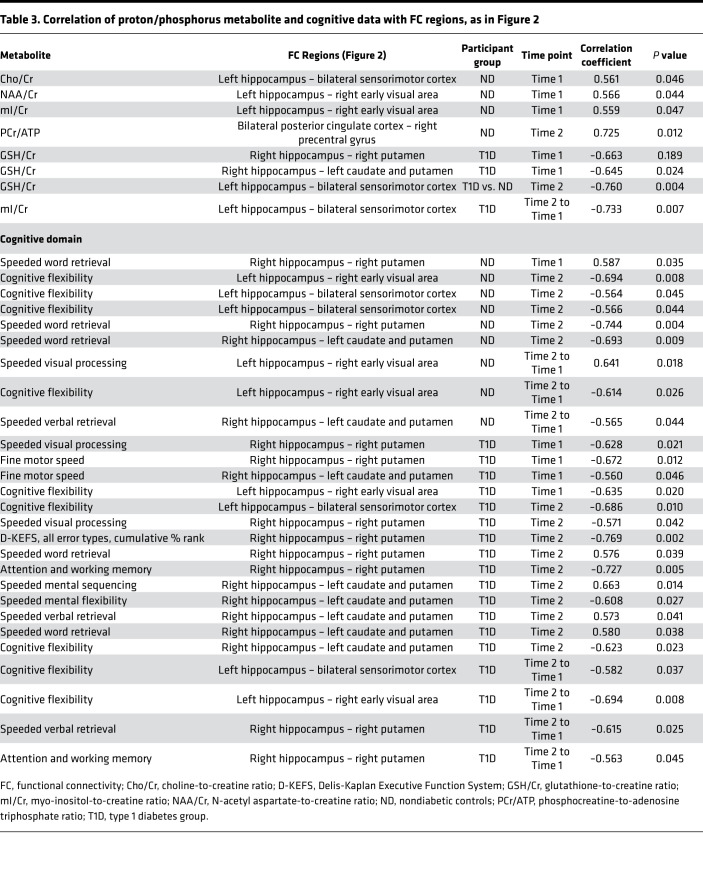
Correlation of proton/phosphorus metabolite and cognitive data with FC regions, as in Figure 2

## Version 1 02/09/2021

In-Press Preview

## Version 2 03/08/2021

Electronic publication
