# Supplementary material for: Brain functions and cognition on transient insulin deprivation in type 1 diabetes
Source: JCI Insight. 2021 Mar 8;6(5):e144014. doi: 10.1172/jci.insight.144014 (PMC8021100; doi:10.1172/jci.insight.144014)
Supplement: Supplemental data [file jciinsight-6-144014-s275.pdf]

Supplement Table 1

AVLT Components - Time 1 (T1) to Time 2 (T2) changes paired t-test

		**Mean ± SEM Recog SS-AVLT**	**Mean ± SEM Trial 6 SS-AVLT**	**Mean ± SEM Total Trials 1-5 SS-AVLT**	**Mean ± SEM Average of List B SS-AVLT**	**Mean ± SEM Delay Recall SS-AVLT**
ND–Adult	T1	104.6 ± 2.5	99.6 ± 6.4	96.9 ± 5.4	93.6 ± 5.1	98.4 ± 5.8
	T2	95.1 ± 7.4	91.6 ± 6.4	84.9 ± 3.7*	92.6 ± 3.5	78.0 ± 8.7*
ND–Adolescent	T1	89.8 ± 6.8	77.3 ± 6.2	78.5 ± 7.9	87.3 ± 4.2	73.1 ± 7.5
	T2	72.5 ± 9.7	61.3 ± 7.0*	59.9 ± 7.3*	87.2 ± 4.3	47.3 ± 4.1*
T1D–Adult	T1	103.1 ± 4.0	95.6 ± 6.5	92.7 ± 4.7	90.7 ± 6.5	95.2 ± 5.9
	T2	87.7 ± 6.5*	94.3 ± 6.5	91.1 ± 5.9	99.7 ± 5.0	81.1 ± 8.6*
T1D– Adolescent	T1	91.9 ± 7.2	77.7 ± 8.8	80.6 ± 7.7	93.2 ± 4.7	77.4 ± 10.6
	T2	47.6 ± 16.7*	64.0 ± 8.8	67.4 ± 9.3	93.4 ± 5.6	34.9 ± 4.6*

*$p<0.05$

Note: There are variable responses in T1D and ND adults and adolescents related to the insulin deprivation and control period. As a result we did not pool the results of adults and adolescents as we have done in other cognitive measurements and reported in Figure 1A.
