# Supplementary material for: Brain functions and cognition on transient insulin deprivation in type 1 diabetes
Source: JCI Insight. 2021 Mar 8;6(5):e144014. doi: 10.1172/jci.insight.144014 (PMC8021100; doi:10.1172/jci.insight.144014)
Supplement: Trial reporting checklists [file jciinsight-6-144014-s276.pdf]

Not applicable
